# Roles of IL-6-gp130 Signaling in Vascular Inflammation

**DOI:** 10.2174/157340308785160570

**Published:** 2008-08

**Authors:** Tieying Hou, Brian C Tieu, Sutapa Ray, Adrian Recinos III, Ruwen Cui, Ronald G Tilton, Allan R Brasier

**Affiliations:** Departments of Biochemistry and Molecular Biology, Internal Medicine, and the Sealy Center for Molecular Medicine, University of Texas Medical Branch, Galveston, TX-77555-1060, USA

**Keywords:** IL-6/ gp-130/ angiotensin II/ STAT3/ vascular inflammation.

## Abstract

Interleukin-6 (IL-6) is a well-established, independent indicator of multiple distinct types of cardiovascular disease and all-cause mortality. In this review, we present current understanding of the multiple roles that IL-6 and its signaling pathways through glycoprotein 130 (gp130) play in cardiovascular homeostasis. IL-6 is highly inducible in vascular tissues through the actions of the angiotensin II (Ang II) peptide, where it acts in a paracrine manner to signal through two distinct mechanisms, the first being a classic membrane receptor initiated pathway and the second, a trans-signaling pathway, being able to induce responses even in tissues lacking the IL-6 receptor. Recent advances and new concepts in how its intracellular signaling pathways operate *via* the Janus kinase (JAK)-Signal Transducer and Activator of Transcription (STAT) are described. IL-6 has diverse actions in multiple cell types of cardiovascular importance, including endothelial cells, monocytes, platelets, hepatocytes and adipocytes. We discuss central roles of IL-6 in endothelial dysfunction, cellular inflammation by affecting monocyte activation/differentiation, cellular cytoprotective functions from reactive oxygen species (ROS) stress, modulation of pro-coagulant state, myocardial growth control, and its implications in metabolic control and insulin resistance. These multiple actions indicate that IL-6 is not merely a passive biomarker, but actively modulates adaptive and pathological responses to cardiovascular stress.

**Summary:**

IL-6 is a multifunctional cytokine whose presence in the circulation is linked with diverse types of cardiovascular disease and is an independent risk factor for atherosclerosis. In this review, we examine the mechanisms by which IL-6 signals and its myriad effects in cardiovascular tissues that modulate the manifestations of vascular inflammation.

## INTRODUCTION

IL-6 is a multifunctional cytokine that has been widely implicated in cardiovascular disease. Produced by a wide spectrum of cell types in the cardiovascular system, IL-6 secretion is upregulated in response to inflammation, angiotensin II (Ang II), oxidative stress and vascular injury [1-3]. Because of its ability to sense cardiovascular stress, IL-6 has become a marker of vascular inflammation, where its increase in the circulation is epidemiologically associated with a variety of clinically significant outcomes. Although IL-6 is clinically considered to be a biomarker of cardiovascular disease, emerging evidence indicates that IL-6 signaling plays a central, significant biological role in cardiovascular regulation. In this review, we will discuss new studies which elucidate its signaling pathways, and implicate its actions in mediating systemic inflammation (hepatic acute phase induction and modification of thrombotic pathways), homeostatic functions (cellular cytoprotection from ROS stress), endothelial dysfunction, cellular inflammation (monocyte activation), growth control (intimal proliferation and cardiac hypertrophy), and metabolic control (insulin resistance). These multiple actions indicate that IL-6 is not merely a passive biomarker, but actively modulates responses to cardiovascular disease.

## IL-6 AND CARDIOVASCULAR DISEASE

Although it is outside the scope of this review to detail all potential roles of IL-6 (or its downstream product, C-reactive protein (CRP)) as a cardiovascular biomarker in different cardiovascular diseases, it is important to emphasize that elevation of IL-6 is associated with diverse pathologies. Increased circulating IL-6 is associated with a number of cardiac risk factors, including atherosclerotic disease, cardiomyopathies, and metabolic syndromes. For example, in seminal observations emanating from the Physicians Health Study, baseline plasma concentration of IL-6 is associated with increasing risk of myocardial infarction (MI). Here, individuals with the highest quartile of IL-6 values have a 2.3-fold increased relative risk of having an MI relative to those with the lowest IL-6 values [4]. Importantly, in this study the IL-6 association remained significant even after adjusting for conventional cardiovascular risk factors. In patients admitted for acute coronary syndromes, increases in circulating IL-6 in the first two days of hospitalization are positively correlated with risk of reinfarction and in-hospital complications [5]. In apparently healthy middle-aged men, multiple measures of blood pressure strongly correlate with circulating IL-6 levels [6]. In congestive heart failure, circulating IL-6 inversely correlates with AHA functional classification, ejection fraction, and survival [7,8]. In studies designed to identify plasma predictors of peripheral arterial disease (PAD), the downstream induced protein of IL-6, CRP, is strongly and independently associated with symptomatic PAD [9]. Additionally, in obesity, serum IL-6 levels are positively correlated with extent of obesity [10,11] and risk for subsequent development of overt diabetes [12]. Together, these observations indicate that circulating IL-6 is a marker for common pathophysiologic processes underlying clinically significant cardiovascular disease.

## CARDIOVASCULAR INDUCERS OF IL-6

IL-6 is highly inducible in response to cytokines (IL-1, TNFα), viral infection, and Ang II [13,14]. Because of its central role in mediating cardiovascular inflammation, the mechanisms by which Ang II activates IL-6 have been intensively investigated. In smooth muscle cells and hepatocytes, Ang II activates IL-6 expression *via* the type 1 Ang II receptor (AT1R) [3]. Studies by our group have shown the essential role of the NF-κB transcription factor in mediating inducible IL-6 expression [13]. NF-κB is a cytoplasmic transcription factor that has been implicated in cardiovascular inflammation and is known to be regulated by several distinct pathways that control its cytoplasmic-to-nuclear partitioning [Reviewed in [15]]. Our recent work has shown that Ang II induces NF-κB *via* an entirely distinct mechanism-one that activates the latent transcriptional activity of the RelA transcriptional subunit, mediated by the Rho family of GTPases. This process culminates in enhancing phosphorylation in the RelA COOH transactivation domain at serine residue 536 and formation of a nuclear complex with the NF-κB inducing kinase (NIK) [16]. IL-6 gene expression results when the activated phosphorylated form exchanges with inactive unphosphorylated RelA bound to the IL-6 promoter [13]. Recent studies from the Lucas laboratory have defined further key signaling intermediates of the Ang II signaling pathway converging on NF-κB [17]. This group has identified a requirement of three additional signaling molecules that form an activated membrane bound complex. These proteins include: (i) CARMA3 [caspase recruitment domain (CARD)], a tissue specific member of the membrane associated guanylate-kinase superfamily of scaffolding proteins, which serves to integrate the upstream signal of activated protein kinase C with downstream factors, (ii) Bcl10, an intermediate bridging factor; and (iii) MALT1, an effector protein that oligomerizes through interaction with Bcl10 [17]. The interaction of the CARMA3/MALT1/Bcl10 complex with the NF-κB signaling pathway is actively under investigation and should provide novel therapeutic targets to selectively disrupt Ang II-induced vascular inflammation without affecting pathways controlling adaptive immunity and cellular apoptosis.

Recent studies indicate that several tissues are affected by enhanced IL-6 associated with vascular inflammation. First, IL-6 has actions locally in the vessel wall. For example, IL-6 production has been identified locally in coronary atherosclerotic plaques [18], where it co-localizes with Ang II [18], as well as aortic atherosclerotic plaque in experimental rodents [2]. In Ang II-stimulated vessels, IL-6 is the most abundantly secreted cytokine detected. Here IL-6 is predominantly expressed by fibroblasts and activated macrophages in the adventitial layer of the proximal ascending aorta, with lesser amounts in the media and intimal layers [2]. Moreover, these studies demonstrated that the IL-6 signaling pathway was locally activated in both adventitial and endothelial layers [2]. These data indicate that Ang II activates a local IL-6 signaling pathway in the aortic adventitia during very early phases of Ang II-induced atherosclerosis.

## MECHANISMS OF IL-6 SIGNALING

### Classical Membrane IL-6 Signaling

IL-6 is the prototype for one of the most pleiotropic cytokine family in mammals, a family that includes IL-11, oncostatin M (OSM), cardiotrophin-1 (CT-1), ciliary neurotrophic factor (CNTF), cardiotrophin-like cytokine (CLC), leukemia inhibitory factor (LIF), and the recently identified IL-27p28 [19-21]. This family contains structures with four long α-helices arranged in an up and down topology. IL-6 is a highly inducible cytokine secreted by several different cell types of cardiovascular relevance, including macrophages, lymphocytes, fibroblasts, endothelial cells and smooth muscle cells [22-26]. Since IL-6 is the major hormonal mediator of the hepatic acute-phase reaction, mechanisms for IL-6 signaling have been intensely studied. Currently we know that IL-6 activates target cells through a classical signaling pathway by binding cell surface IL-6 receptor α-subunits (IL-6Rα).

**Fig. (1).**
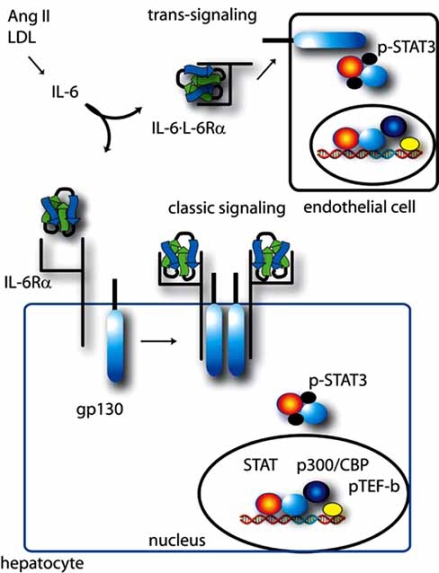
**IL-6 induced classical and trans-signaling pathways.** Shown is a schematic view of classical IL-6 signaling *via* the IL-6Rα receptor and gp130 for a representative hepatocyte. IL-6Rα bound to the IL-6 ligand results in complex formation with gp130, activating tyrosine kinase activity, including and culminating in tyrosine phosphorylation of STAT3. The IL-6 trans-signaling pathway is diagrammed at top, using a representative endothelial cell. Circulating IL-6∙IL-6Rα engages with gp130 expressed on cells, enabling activation of the IL-6 signaling pathway in cells lacking IL-6Rα. See text for further details.

Molecular events in IL-6 signaling are initiated by binding to its receptor subunit IL-6Rα (which has no intrinsic kinase activity) with low affinity at the cell surface. The IL-6∙IL-6Rα complex then triggers ligand-mediated oligomerization with the ubiquitously expressed transmembrane gp130 β-subunit, inducing gp130 homodimerization, and subsequent formation of a hexameric IL-6∙IL-Rα∙gp130 high-affinity complex [27] (Fig. **1**). Receptor ligation induces conformational changes in the cytoplasmic domains of gp130 that bring Janus tyrosine kinases (JAKs) into close proximity. This molecular interaction results in trans-autophosphorylation of JAK1, a specific Janus kinase mediating IL-6 signaling [21,28,29]. JAK1, in turn, phosphorylates gp130 on the docking sites for the signal transducer and activator of transcription (STAT); STAT isoforms -1 and -3 are then recruited, where they, too, become phosphorylated [21]. In addition to STAT activation, phosphorylation of gp130 on membrane proximal Tyr 759 residue is necessary and sufficient for binding of SRC homology domain 2-containing tyrosine phosphatase 2 (SHP-2). SHP-2 is then phosphorylated, and by itself or together with another docking protein, Grb2 (growth factor receptor binding protein 2)-associated binder-1 (Gab1), and initiation of the Ras-ERK-MAPK cascade occurs [19,21,28,30].

Of the signaling pathways downstream of the IL-Rα∙gp130 complex, STAT appears to play a major role. Tyr phosphorylated STATs-1 and -3 then form intermolecular associations, homo- and hetero-dimerize and translocate into the nucleus, where they bind specific DNA sequences (for example, acute phase or IL-6 response elements) and enhance transcription of target genes [21,29,31]. Analyses of complex formation with STAT3-dependent transcriptional enhancers have shown that STAT3 undergoes additional post-translational modifications that permits interactions with co-factors and co-activators [32] .

**Fig. (2).**
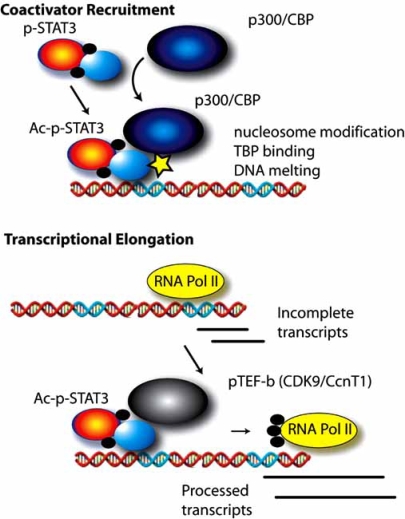
**Discrete mechanisms for IL-6 induction of target genes.** Top, coactivator recruitment mechanism. Tyrosine phosphorylated STAT3 binds to p300/CBP, resulting in STAT3 acetylation (Ac) on its NH_2_ terminus, and stabilization of the STAT3-p300/CBP complex. The acetylated-phosphorylated STAT3-p300/CBP complex then binds to high affinity IL-6 response elements in the promoters of target genes. This complex induces nucleosomal reorganization *via* the p300 histone acetylase activity, pre-initiation complex formation, recruiting TATA box binding protein, and enhanced RNA polymerase II activity. Bottom, transcriptional elongation. In a subset of IL-6 responsive promoters, RNA polymerase (Pol) II is engaged with the promoter producing incomplete transcripts. During the process of activation, tyrosine phosphorylated STAT3 complexes with the positive transcriptional elongation factor (PTEF-b), a complex containing CDK9. CDK9 phosphorylates the COOH terminal domain of RNA polymerase II, enabling it to enter productive elongation mode, producing full length RNA transcripts.

Recent studies have shown that STAT activities are modulated by their interactions with co-factors which positively or negatively regulate their activity. For example, upon entry into the nucleus, STAT3 associates with the p300/ CREB-binding protein (CBP) coactivator, an enhancer protein with intrinsic histone acetyltranferase (HAT) activity which is able to open chromatin structure, allowing other chromatin-modifying proteins to bind to DNA and activate transcription [33-35] (Fig. **2**). The p300/CBP association requires both the NH_2_-terminal modulatory domain and the COOH-terminal transactivation domain of STAT3 [35,36]. Interestingly, STAT3 itself can also be acetylated by p300/ CBP at these two domains in response to IL-6. Acetylation on Lys 685 on its COOH-terminal region is critical for stable dimer formation and DNA-binding activity [36]. Studies from our laboratory first described two novel acetylation sites on the STAT3 NH_2_ terminus at Lys 49 and -87 that are required to stabilize the STAT3-p300/CBP complex through an additional interaction mediated by the modified STAT3 NH_2_ terminus [37].

Further, we have recently discovered that STAT3 also regulates downstream gene expression by promoting transcription elongation [38] (Fig. **2**). This function is realized by the interaction between STAT3 and Positive Transcription Elongation Factor (PTEF-b) [38,39], a complex that phosphorylates Ser 2 on the heptad repeat of the COOH terminal domain of RNA polymerase II. COOH terminal phosphorylation permits Pol II to escape from transcription arrest and produce full length mRNA transcripts. Specifically, we found that activated nuclear STAT3 forms complexes with Cyclin-Dependent Kinase 9 (CDK9), the major component of PTEFb, through both its NH_2_- and COOH-terminal domains. STAT3 binding then results in CDK9 being recruited to the promoter and downstream coding region of target genes. Importantly, induction of STAT3 target genes, such as γ-FBG and p21*waf1*, are significantly reduced when CDK9 kinase activity is inhibited [38], suggesting that it is possible to modulate cellular response induced by the IL-6-STAT3 pathway by targeting CDK9 (Fig. **2**).

### IL-6 Trans-Signaling

Recently it has been observed that the IL-6 tissue response is significantly enhanced by a phenomenon termed “trans-signaling” [29,31] (Fig. **1**). This mechanism is suggested by the findings that the IL-6Rα also exists in a soluble plasma form lacking the transmembrane domain, and that membrane association of the IL-6Rα subunit with gp130 is not required for signal initiation. Although soluble IL-6Rα can be formed by alternative splicing of the receptor transcript, the chief means of its production is through ectodomain shedding from activated leukocytes by a disintegrin and metalloproteinases (ADAMs)-17 and -10 [26,29,40,41]. Although the membrane-associated IL-6Rα is specific to only certain cell-types, gp130 is ubiquitously expressed. Thus, the soluble IL-6-IL-6Rα complex can initiate IL-6 signaling on any cell type. This of course, expands the repertoire of IL-6 responsive cells to virtually any cell in the body [29].

Also recently, it has been shown that IL-6 signaling in inflammatory disease utilizes classic or trans-signaling mechanisms to different degrees depending on specific cell-types and pathologies. There also exists a little understood and naturally occurring soluble form of gp130 [29,31]; this protein can be used as a tool to differentiate classic cell surface IL-6 signaling versus trans-signaling processes because soluble gp130 competitively inhibits trans-signaling without affecting membrane-bound signaling. In one study, administration of soluble recombinant gp130 in a rodent model demonstrated that Ang II-dependent hypertension required IL-6 trans-signaling, but concomitant vascular hypertrophy, down-regulation of the AT1R, and STAT3 activation were responses mediated through classic cell surface IL-6R signaling [42]. In another elegant study, using a transgenic mouse overexpressing soluble gp130, effectively inhibiting IL-6 trans-signaling [43], mononuclear cell-dominated inflammatory processes were selectively inhibited, indicating that mononuclear inflammation relied on trans-signaling rather than classic signaling. Further, some inflammatory processes in these mice were blocked to the same degree as in an IL-6 knockout mouse [43]. These approaches hold promise to elucidate these two mechanisms of IL-6 signaling in various pathological processes.

### Negative Regulation of IL-6 Signaling

**Fig. (3).**
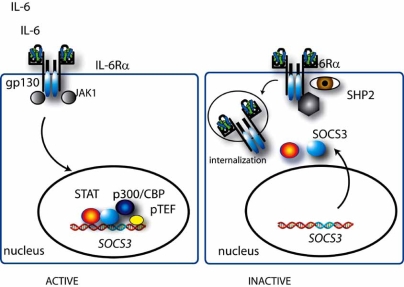
**Negative regulation of the JAK-STAT pathway.** Shown are the major negative autoregulatory pathways of IL-6 induced STAT3 signaling. IL-6 activated STAT3 both engages the suppressor of cytokine signaling (SOCS3) gene, inducing its expression and recruits SOC3 to gp130 where it subsequently terminates STAT3 activation *via* JAK1 inactivation. SHP2 phosphatase activity also inactivates gp130 and JAKs.

Several negative feedback mechanisms provide temporal control of IL-6 signaling (Fig. **3**). Ligand-induced internalization and degradation of IL-6Rα and gp130 has been identified as a proximal mechanism for negating signaling [31]. IL-6 signaling is particularly sensitive to the downstream STAT3-dependent recruitment of suppressor of cytokine signaling 3 (SOCS3) to the gp130 Tyr 759 residue, a site near where JAK1 binds. SOCS3, itself inducible by STAT3, inhibits JAK1 activity through an unknown mechanism involving its kinase inhibitor domain [21,44,45]. IL-6-gp130 signaling is also attenuated by a phosphorylation-dependent induction of SHP-2 tyrosine phosphatase activity which dephosphorylate gp130 and JAKs [19,46]. Finally, SOCS proteins have been observed to recruit the elongin BC ubiquitin-ligase complex to JAKs, and perhaps other components of the receptor complex, promoting ubiquitination and subsequent proteosomal degradation [21,28,44]. Together these inhibitory mechanisms ensure transient IL-6 action.

## CELLULAR TARGETS AND ACTIONS OF IL-6

### Endothelial Cells

Enhanced ROS stress has been implicated in the pathogenesis of atherosclerosis, hypertension, diabetes, aging and mechanical injury [47-52]. ROS are either oxygen-centered free radicals that include superoxide (O_2_^-^), hydroxyl radicals (^.^ OH) and lipid (L) hydroperoxides (LOO), or reactive non-radical compounds that include (H_2_O_2_), singlet oxygen (^1^O_2_), hypochlorous acid (HOCl) and chloramines (RNHCl) [53,54]. Under normal conditions the rate and magnitude of oxidant formation is balanced by the rate of oxidant elimination (an enzymatic activity influenced by IL-6). However, oxidant overproduction produces an imbalance that over-whelms cellular antioxidant capacity, damaging cellular lipids, membranes, proteins and DNA [55,56]. In addition, ROS can act as second messengers in an autocrine or paracrine fashion to modulate endothelium-dependent vasorelaxation, smooth muscle cell and endothelial cell growth and survival, and vascular remodeling. Each of these responses, when uncontrolled, contributes to vascular disease [57].

Because of these important activities of ROS, the mechanisms by which they are generated have been extensively investigated [49,58]. Although macrophages are the major source of most ROS in the vessel wall, other cells, such as endothelial, smooth muscle and adventitial cells, produce ROS. Ang II and lipid (oxidized) LDL appear to be potent inducers of ROS [59]. In fact, Ang II infusion doubles O_2_^-^ production in aortic segments [49]. As a result, during the early stage of Ang II-induced atherosclerosis, the nonadhesive function of endothelium which controls vasomotor tone, is disturbed. This process is mediated by Ang II–induced production of ROS, which results in the chemical inactivation of nitric oxide (NO), blunting its ability to vasodilate [60]. Among the variety of ROS generators in VSMCs are the mitochondrion and cellular enzymes, such as xanthine oxidase, cyclooxygenase, lipoxygenase, NO synthase, heme oxygenases, peroxidases, and the membrane-associated NAD(P)H oxidases, the latter having been shown to be of foremost physiological importance [58]. Inhibition of vascular ROS production through administration of superoxide dismutase increases acetylcholine-induced relaxation, suggesting that ROS species themselves are responsible for the endothelial dysfunction [49]. In humans, it has been shown that treatment with selective inhibitors of AT1R reverses endothelial dysfunction in large arteries [28].

Because IL-6 upregulates AT_1_R gene expression, it may lead to increased Ang II-mediated vasoconstriction and ROS production, and thereby play an important role in mediating endothelial dysfunction [61]. Consistent with this idea, it was observed that IL-6 deficiency protects against Ang II – induced endothelial dysfunction [62]. This IL-6 effect occurs locally within the vessel wall, independent of increases in blood pressure. Schrader *et al.* have reported that an O_2_^-^ scavenger restored endothelial responses in Ang II-treated arteries [62]. These findings further suggest that the effect of Ang II on endothelial function is attributable to O_2_^-^-mediated inactivation of NO [49,63]. Importantly, Ang II increases in ROS tone are absent in mice deficient in IL-6 or Nox2 genes. Together, these data suggest that NAD(P)H oxidase is a major source of O_2_^-^ and a primary mediator of endothelial dysfunction. Thus, IL-6 may be a critical link in NAD(P)H-derived, O_2_^.-^-mediated impairment of NO-induced vascular relaxation. Whether activation of NAD(P)H oxidase by Ang II occurs upstream or downstream of IL-6 expression remains unclear. IL-6 expression may be an important link between Ang II–induced increases in NAD(P)H oxidase activity, limiting the bioavailability of NO for normal vascular responses.

Interestingly, endothelium does not express transmembrane IL-6Rα and is unresponsive to IL-6; however, endothelium can be activated by the IL-6 trans-signaling pathway discussed earlier [64]. IL-6 trans-signaling may play important roles in other endothelial-dependent functions. For example, in addition to vasodilation, the endothelium also plays a central role in regulating hemostasis, expressing anti-coagulant and anti-adhesion molecules [65]. Both acute vascular inflammation and chronic injury can cause inappropriate activation of endothelium, converting it to a prothrombotic surface [66]. The actions of endothelial cells on haemostasis are tightly regulated by a network of cytokines in autocrine or paracrine mechanisms [67]. Inflammatory stimuli, such as lipopolysaccharide (LPS) or cytokines, can activate endothelial cells, resulting in the synthesis of IL-1, IL-5, IL-6, IL-8, IL-11, IL-15; as well as colony-stimulating factors and chemokines. These secreted cytokines not only affect the local microenvironment by inducing local inflammation and thrombosis, but also influence the systemic inflammatory responses and global hemostatic balance. Specific inducers of IL-6 production by vascular cells include IL-1 [64], LPS [68], TNFα [69] and IL-4 [70]. Co-stimulation of endothelial cells with IL-4 and interferon-γ or IL-4 with IL-1 further amplify the synthesis of IL-6 mRNA [70,71]. Interactions between IL-6 and endothelial cells regulate recruitment of leukocytes and expression of chemokines. IL-6^-/-^ mice show defective leukocyte accumulation to inflammatory sites, which is associated with decreased synthesis of chemokines by endothelial cells and reduced surface expression of adhesion molecules [72]. Cultured endothelial cells (HUVEC) have been shown to produce MCP-1, -3, IL-8, as well as IL-6. Upregulation of intercellular adhesion molecule-1 (ICAM-1) also is observed in the presence of a trans-signaling complex, IL-6·IL-R, at physiological concentrations [72]. The multiple inducers mentioned above promote interactions between endothelial cells and leukocytes, platelets or red blood cells, leading to further activation and damage of endothelium in an autocrine amplification pathway.

### Monocytes

Macrophages play an important role in vascular inflammation where they locally secrete cytokines, chemokines, and matrix metalloproteinases that promote further cellular infiltration and vascular remodeling. Vascular macrophages are derived from peripheral blood monocytes which locally differentiate into macrophages. This is a complex, multi-step process; importantly, IL-6 is a prominent cytokine that promotes monocyte-to-macrophage differentiation.

IL-6 promotes macrophage differentiation, growth arrest and eventual apoptosis *in vitro* [73-79]. Experiments using IL-6 stimulation of myeloid leukemia cell lines have shown that IL-6 induces development of mature macrophages [73,74,80,81]. IL-6 stimulation causes these cells to increase in size, develop a large vacuolar cytoplasm, develop irregularly shaped nuclei [74-76,82] and become surface adherent [77]. Functionally, these cells have increased esterase and phagocytic activities [74]. Surface expression of C3 complement receptors, Fc receptors, and macrophage-colony stimulating factor (M-CSF) receptors along with F4/80, a marker for mature macrophages, also are up-regulated [76, 77,83]. CD36, an oxidized lipid uptake receptor, has been shown to be induced by IL-6 in mouse peritoneal macrophages [84]. Furthermore, IL-6 induces expression of genes typical of macrophages, including the early response genes c-Jun, jun B, jun D, interferon-regulatory factor 1 (IRF1), JAK3, and Egr-1 [74,76,77,79]. However, expression levels of c-myc mRNA go down within hours of IL-6 stimulation, facilitating growth arrest and differentiation [74,77-79]. Bcl-2 and cyclin D1 are down-regulated subsequently, increasing susceptibility to apoptosis [76,85]. IL-6 also regulates late response genes, including lysozyme and ferritin light-chain, genes that are normally induced during terminal macrophage differentiation [77,86]. Moreover, upon stimulation with IL-6, monocytic cells up-regulate MCP-1 mRNA and protein, a chemokine more strongly expressed in macrophages than monocytes [87].

Additional work has shown that IL-6 favors monocyte-to-macrophage differentiation rather than monocyte to dendritic cell differentiation [83,88,89]. Monocytes cultured in the presence of IL-4 and GM-CSF become CD1a^+^CD14^-^ dendritic cells (DCs), but the addition of IL-6 alone causes CD1a^-^CD14^+^ macrophage differentiation, [88], where morphological and functional characteristics of macrophages are seen [83,88]. Interestingly, this effect is not seen with the addition of other IL-6 family member cytokines, including IL-11, LIF, and OSM; monocytes become dendritic cells in their presence [88]. The favoring of macrophage differentiation by IL-6 is also seen in monocyte and fibroblast co-culture where the cell-cell interaction is thought to induce large amounts of IL-6 [83]. Even in the presence of IL-4 and GM-CSF in this system, the dendritic cell differentiation program is overridden by IL-6 signaling, and monocytes develop into macrophages [83]. This may be due to the observation that IL-6 up-regulates the number of functional M-CSF receptors on moncytes, thereby increasing their sensitivity to M-CSF [83].

gp130 and downstream signaling is required for IL-6-induced macrophage differentiation [79]. Expression of gp130 mutations that are unable to activate STAT3 prevents the subsequent growth arrest and differentiation of M1 myeloid cells [79]. Furthermore, downstream STAT3 activation is required because dominant-negative forms of STAT3 block differentiation [78,90]. Specifically, Minami *et al.* showed that inhibiting STAT3 prevented induction of Fc receptors, ferritin light chain, and lysozyme; moreover, c-myc was not down-regulated and the cells continued to proliferate [78]. Likewise, over-expression of JAK3, which also is induced rapidly by IL-6, accelerates macrophage differentiation [91]. Therefore, IL-6 activates the gp130-JAK/STAT signaling pathway leading to differentiation of monocytes.

Other transcription factors have been shown to modulate the IL-6-induced macrophage differentiation phenomenon. GATA-1, an erythroid nuclear protein that regulates globin gene expression, inhibits IL-6-induced macrophage differentiation and apoptosis [76]. Overexpression of GATA-1 in M1 cells leads to megakaryocytic or erythroid differentiation even in the presence of IL-6 and normal STAT3 signaling [76,92]. Tanaka *et al.* reported that expression of bcl-2 and cyclin D1 in these cells remain sustained, thereby disrupting the effect of IL-6 [76]. Thus, although the STAT3 pathway is necessary for IL-6-induced macrophage differentiation, altered gene transcription induced by other pathways can modulate this process. In contrast to GATA-1, the zinc finger transcription factor Egr-1 is thought to be essential for macrophage differentiation [77,93]. Knocking down Egr-1 with anti-sense oligonucleotides blocks M1, HL-60, and U-937 myeloid cell lines from differentiating into morphologically mature macrophages while overexpression of Egr-1 leads to activation of macrophage differentiation even without the presence of IL-6 [77,93]. Increased Egr-1 activity, by itself, decreases growth rate and expression of c-myc, increases Fc and C3 receptors, and elevates expression of jun B, ferritin light-chain, and lysozyme [77]. Simulation with IL-6 accelerates this process, resulting in increased cell adherence to culture dishes and a doubling of the percentage of morphologically-mature macrophages as compared to Egr-1 over-expressing cells alone [77]. However, the lack of Egr-1 in mice does not impair macrophage differentiation and activation *in vivo* [94]. This might be due to the possibility that other members of the Egr-1 family (Egr-2,-3, and-4) have redundant activities [77].

Despite the wealth of *in vitro* data, there is little evidence that IL-6 plays a critical role in macrophage differentiation *in vivo*. Macrophages from IL-6 deficient and wild-type mice are similar, and there is no report on decreased numbers of mature macrophages in the IL-6 deficient mouse. Peritoneal macrophages from IL-6 knockout mice express levels of major histocompatibility complex (MHC) class II and F4/80 comparable to wild-types, and they can be stimulated *in vitro* by LPS and IFN-γ to produce almost equal amounts of nitric oxide [95,96]. However, it is well known that IL-6 deficiency results in impaired protection against bacterial infections *in vivo*, particularly to *Listeria monocytogenes* [96-99]. *L. monocytogenes* is a bacterium that primarily infects and proliferates intracellularly in macrophages; activation of macrophages is required to destroy the bacterium. Interestingly, IL-6 deficient mice fail to control infections by *L. monocytogenes* [95-97]. Bluethmann *et al.* have proposed that this reduced anti-bacterial defense might be due to a defect in differentiation of macrophages in bone marrow [98]. In fact, the number of myeloid progenitors (CFU-GM) that give rise to granulocytic-monocyte lineage are reduced by half in the bone marrow of IL-6 deficient mice and are increased 4-fold in the spleen [100]. Although the total number of CFU-GM does not increase overall, the redistribution from bone marrow to the spleen may affect the differentiation and/or function of the macrophages. Except for the decreased ability to fight infections, there are no other reported differences in macrophages obtained from IL-6 deficient mice. Thus, other factors that promote macrophage differentiation may compensate for the lack of IL-6. IL-6 might be sufficient, but it does not seem to be necessary for macrophage differentiation *in vivo*.

Nevertheless, IL-6 probably contributes to macrophage differentiation in vascular inflammation where IL-6 levels are highly elevated in serum and cardiovascular tissues. In Ang II-infused mice, IL-6 secretion in aortic tissue is increased 4-fold over basal levels [2]. The location of IL-6 production and the site of activated STAT3 is predominantly in the adventitia, a location, coincidently, where the majority of macrophages reside [2,101]. This strong co-localization suggests that the monocytes recruited into the adventitia are most likely stimulated by the IL-6 present, along with other pro-macrophage factors, to become macrophages. Although not directly studying macrophages recruited to vascular tissue, Keidar *et al*. reported that CD36 was up-regulated on peritoneal macrophages in association with increased IL-6 serum levels in Ang II-infused mice [84]. CD36 was further shown to be directly inducible by IL-6 [84]. This study suggests that IL-6 may promote macrophage differentiation in vascular tissue by up-regulating CD36 on recruited monocytes. Clearly, more research is needed to elucidate the roles of IL-6 in differentiating monocytes to macrophages in vascular tissue itself. 

### Platelets

Platelets are not only essential for blood coagulation, but also for modulating inflammatory processes and contributing to wound healing by producing cytokines, chemokines, growth factor and other inflammatory mediators [102]. IL-6 is known as an important regulator of megakaryocyte differentiation and maturation *in vitro* [103,104], particularly when stimulated in conjunction with IL-1α and IL-3 [105]. Several *in vivo* studies provide evidence that IL-6 induces thrombocytosis. It has been shown that recombinant IL-6 and other IL-6 family members, including IL-11, OSM, and LIF, significantly enhance peripheral blood platelet count in experimental animals [106-109]. The relationship between IL-6 and thrombocytosis also was observed in a correlative study in which 83 % of patients with secondary thrombocytosis had elevated serum IL-6 levels [110]. IL-6 not only augments platelet count, but also affects platelet function. IL-6-treatment enhances platelet responsiveness to thrombin stimulation and increases P-selectin expression, a sensitive marker of platelet activation [111,112]. Also, incubation of platelets with IL-6 *in vitro* caused a dose-dependent enhancement of agonist induced maximum aggregation (AIMA) and secretion of thromboxane B2 (TXB2), indicating the activation of platelets [113,114]. Involvement of arachidonic acid metabolism is suggested since both AIMA and TXB2 production were inhibited by indomethacin and dazoxiben [113].

### Myocardiocytes

Although the individual roles of the IL-6 family of cytokines are not fully elucidated, the gp130-signaling pathway is known to play a key role in hypertrophic response of the myocardium to acute pressure overload. For example, genetic modifications that result in tonic gp130 activation produce cardiovascular hypertrophy in mice [115]. Because organism-wide knock-out of gp130, STAT3, LIF or CT-1 is embryonically lethal with exhibition of multiple organ defects, the cardiovascular role of these molecules has been difficult to discern. The development of cre-lox technology for tissue-specific gp130 knockout in ventricular muscle resulted in the surprising observation that gp130 signaling was not required for cardiac development or baseline indices of cardiac function [116]. However, when these animals were challenged in a model of acute pressure overload by aortic banding, instead of compensatory hypertrophy, gp130 knockout mice rapidly showed signs of cardiac failure, including reduced fractional systolic shortening and increased LV end diastolic pressure, followed later by development of dilated cardiomyopathy with increased cardiomyocyte apoptosis [116]. Other studies have also found that the JAK-STAT3 pathway is activated in acute MI, where the greatest induction appears at the border between the infarct and viable tissue, and inhibition of signaling produces increased myocardial apoptosis [117]. Together, these studies indicate that the gp130 signaling pathway mediates the interface between compensatory hypertrophy and cardiomyocyte apoptosis in response to pressure overload and ischemic insults. Because of the multiple redundant actions of the IL-6/LIF/OSM/CT-1 cytokine family, the individual cytokines that mediate the early gp130 activation have not been definitively determined.

### Hepatocytes

IL-6 is a key effector cytokine in hepatic physiology, including inducing hepatoprotection, mitogenesis, and the acute phase response [118,119]. The acute phase response has been extensively reviewed elsewhere [1,120] and will not be further discussed here. IL-6 has recently been shown to modulate hepatocyte survival. For example, the cell death-inducing ligand, Fas, and toxin-mediated liver injury produce direct mitochondrial damage, ROS generation and subsequent hepatocyte necrosis (with a lesser degree of apoptosis). IL-6 signaling *via* STAT3 induces both antioxidant Ref-1 and caspase inhibitors, including Bcl-2, FLIP, and Bcl-XL, to induce hepatoprotective state [121]. Separately, IL-6 also promotes liver regeneration, to restore liver mass after necrotic or apoptotic injury has occurred.

Haga *et al.* has identified ROS as a component of Fas-mediated liver injury and identified an endogenous antioxidant, Ref-1, as a target of STAT3 [121]. Expression of Ref-1 provided hepatoprotection, strongly suggesting that Ref-1 is a critical component of STAT3-mediated hepatoprotection. Ref-1, a dual-function protein upregulated by increases in ROS, is an endonuclease in the base excision repair pathway and a reducing agent that facilitates the DNA-binding properties of redox-sensitive transcription factors [122,123]. Ref-1 is able to suppress ROS generation and hepatic apoptosis.

A recent study demonstrated that IL-6 also has a protective effect on Fas-mediated liver injury similar to the effect seen with STAT3 by upregulating c-FLIP, Bcl-2 and Bcl-xL [124]. The data of Haga *et al*. implicate that this effect of IL-6 is mediated by STAT3 [121]. More work will be required to dissect the inter-relationships between Ref-1 and STAT3 in IL-6 signaling and cellular survival.

Of the myriad hepatic proteins that IL-6 induces with cardiovascular activities, it is increasingly clear that IL-6 is a major modulator of the coagulation pathway [125-127], where its actions shift hemostatic balance to prothrombosis, thereby increasing the risk of cardiovascular diseases. IL-6 promotes coagulation by a number of mechanisms. First, it increases the expression of procoagulant factors, such as fibrinogen (FBG), tissue factor (TF) and factor VIII [128-130], and reduces the production of antithrombotic factors, such as antithrombin and protein S [112,131]. Second, as discussed earlier, IL-6 is involved in the activation of endothelial cells and plays a central role in hemostasis [67,132]. Third, IL-6 contributes to thrombosis by increasing platelet numbers and regulating their functions [133].

Fibrinogen (FBG) is a large glycoprotein consisting of three pairs of non-identical polypeptides (Aα, Bβ, and γ) which are encoded by separate genes [134]. It is not only a rapid and sensitive marker of the acute phase response, but also an important mediator of hemostasis by participating in clot formation, platelet aggregation and clot retraction [135]. IL-6 can stimulate mammalian hepatocytes to produce FBG in a dose-dependent manner [128]. IL-6 response elements have been identified in the promoter regions of all human FBG Aα, Bβ, and γ genes [136-138]. Analysis of the 5’-flanking region of human FBG Aα identified six potential IL-6 responsive sequences, among which a single sequence of CTGGGA localized from -122 to -127 bp is a functional element [136]. Also, a CCAAT/enhancer binding protein site (C/EBP, -134 to -749 bp) was found adjacent to the functional IL-6 response element (IL-6RE), which might modulate and further increase the magnitude of IL-6 response [136]. In addition, a hepatocyte nuclear factor 1 (HNF-1) binding site, present from -47 to -59 bp, also was essential for the expression of the human fibrinogen Aα gene [136]. A similar finding was observed in the promoter of the human FBG Bβ gene. The identified DNA sequences essential for full IL-6-induced expression of fibrinogen Bβ included three distinct cis-acting DNA elements: an HNF-1 site at ~85 bp upstream of the transcription start site; a C/EBP binding site between nucleotides -124 and -133; and an IL-6 responsive element (IL-6RE) present just 4 bp upstream of the C/EBP consensus binding site [138-140]. The γ chain of fibrinogen (γ-FBG) plays a crucial role in fibrinogen function by inducing platelet aggregation and leukocyte recruitment in inflammation [141,142], concentrating growth factors and cytokines for wound healing [143-145], and mediating fibrin clot formation. Consequently, transcriptional control mechanisms regulating inducible γ-FBG expression have been extensively investigated. These studies have shown that three IL-6 REs are found in the promoter region of the γ-FBG gene [137,146]. Although all of them contributed to the full promoter activity induced by IL-6, one site (site II) was the major functional IL-6 responsive site [137,146]. Further studies using gel mobility shift assays have shown that the binding affinity of STAT3 to these three elements inversely correlated with their functional activities [147]. In contrast to Aα and Bβ-FBG genes, the promoter activity of γ-FBG was not affected by overexpression of C/EBPβ and C/EBPδ isoforms [137]. Recent findings from our lab indicate that γ-FBG expression in hepatocytes also is regulated by the interaction between STAT3 and coactivators p300/CBP [36,37] and CDK9 [38]. Considering the key role of FBG in blood coagulation, IL-6 exerts its effects on hemostasis by regulating the levels of FBG in circulation.

Tissue factor (TF) is well known for its primary role in the initiation of the extrinsic pathway of coagulation. After vessel injury, TF forms a complex with factor VIIIa, which promotes the activation of factor V, leading to thrombin generation, fibrin deposition and finally clot formation [148]. A two-year follow-up study of 120 patients with congestive heart failure (CHF) revealed a strong correlation between TF and IL-6 levels, suggesting a close link between inflammation and thrombogenesis in CHF [149]. Also, patients with CHF and high IL-6 and TF levels have a poorer prognosis, raising the possibility that IL-6 contributes to the prothrombotic state in CHF through its affects on TF expression [149]. In human mononuclear leukocytes, recombinant IL-6 rapidly induces TF mRNA and protein expression [150]. Also, IL-6 induces an increase in TF surface expression on monocytes, and the upregulation of TF is accompanied by an enhanced monocyte procoagulant activity (PCA) [150]. The actions of IL-6 on TF expression also may be indirectly mediated by CRP, an acute phase reactant that is markedly induced by IL-6 [22]. Highly purified human CRP causes a significant increase of TF mRNA after 4 hours of stimulation and a 75-fold increase in the PCA of human peripheral blood monocular cells that is dependent on TF protein synthesis [129]. In a study of 106 outpatients with atrial fibrillation (AF), it was reported that AF patients have higher levels of IL-6, CRP, TF, and plasma viscosity compared with controls, and significant correlations were reported between these inflammatory markers (IL-6, CRP) and prothrombotic plasma markers [151].

Plasminogen activator inhibitor-1 (PAI-1), the major physiologic inhibitor of fibrinolysis, is also induced by pro-inflammatory cytokines, including IL-1 and IL-6 [152,153], in the human hepatoma cell line, HepG2. Incubation of HepG2 cells with IL-1 caused a rapid and dramatic increase in PAI-1 mRNA expression in a dose-dependent manner. IL-6 alone only had a modest effect on PAI-1 mRNA synthesis, however, when combined with IL-1, a significant accumulation of PAI mRNA was observed [153]. A specific region (-239 to -210 bp) of the PAI-1 promoter was shown to be necessary for IL-1β-inducible expression and mediated the combined induction by IL-1β and IL-6 [154,155]. An increased in the binding activity of C/EBPδ to PAI-1 promoter was induced by either IL-1β or IL-6 stimulation. Downregulation of PAI-1 induction by siRNA against C/EBPδ confirmed the critical role of C/EBPδ in the inducible PAI-1 expression [155]. Both IL-1β and IL-6 increase C/EBPδ mRNA expression [154,155]. Although C/EBPδ functions as a common mediator of PAI-1 expression in IL-1β and IL-6 signaling, different upstream kinases were involved. Activation of C/EBPδ by IL-1β required all three of the mitogen-activated protein kinase pathways (MAPK), while JAK signaling contributed to IL-6-inducible expression of PAI-1 [155]. Interestingly, the HMG-CoA reductase inhibitor (mevastatin) abrogated PAI-1 production induced by IL-1 and IL-6, which was mediated by decreasing the levels of C/EBPδ mRNA and protein, as well as by inhibiting C/EBPδ binding to PAI-1 promoter [154,155]. These findings suggest that statins prevent vascular inflammation, at least in part, by inhibiting C/EBPδ-induced PAI-1 expression.

IL-6 also contributes to a pro-coagulant state by reducing synthesis of antithrombotic proteins. For example, IL-6 negatively regulated the production of antithrombin, a potent inhibitor of coagulation, both *in vivo* and *in vitro* [131]. Protein S, a cofactor to Protein C in the inactivation of factors Va and VIIIa, is another IL-6-regulated anti-coagulant. The function of protein S is regulated by forming an inactive complex with complement protein C4b [156]. In a canine model, exogenous IL-6 significantly decreased levels of free protein S, which recovered to normal levels after the cytokine exposure was discontinued [112].

### Adipocytes

The incidence of obesity and its co-morbidities has increased dramatically worldwide, and is characterized by systemic inflammation with an important, central role for IL-6. Visceral obesity is an independent risk factor for numerous chronic cardiovascular diseases, including atherosclerosis, arterial hypertension, renal glomerulopathies with proteinuria, and diabetes. Diabetes is a major risk factor for premature cardiovascular, cerebrovascular, and peripheral vascular disease. Over the last decade, our understanding of fat tissue has changed dramatically from a simple energy storage organ to an important endocrine organ modulating appetite, energy expenditure, insulin sensitivity, metabolism, endocrine and reproductive systems, inflammation, and immunity [86,157-161]. These effects are mediated by adipocytokines, including leptin, resistin, adiponectin, visfatin, as well as more classical cytokines, including TNF-α, MCP-1, and IL-6. Since adipose tissue is a major source of IL-6, it is generally considered an adipokine. These adipokines act on immune cells, leading to local and systemic inflammation, as well as on vascular cells, leading to obesity-related disorders associated with the metabolic syndrome (including hypertension, atherosclerosis, insulin resistance), diabetes, and cancer [86].

Serum IL-6 levels are positively correlated with extent of obesity based on body mass index [10,11]. Visceral adipocytes harvested from severely obese, nondiabetic patients produce substantially more IL-6 than subcutaneous adipocytes harvested from the same individual. This finding partly explains the relationship between visceral adipose fat deposits and the increased risk of cardiovascular disease in humans. Interestingly, visceral adipocytes account for only 10 % of total adipose tissue production of IL-6 [162]. The remainder is produced by non-adipose stromal cells, vascular endothelial cells, and monocyte/macrophages [162,163]. Nevertheless, adipose tissue contributes significantly to the serum pool of IL-6, and the observation that the venous drainage of omental adipose tissue flows directly into the liver suggests an important metabolic impact, particularly on VLDL secretion and hypertriglyceridemia, which could impact on atherosclerosis. In addition, it is well appreciated that IL-6 induces hepatic CRP production, which is an independent risk marker of cardiovascular disease [164,165]. It has been estimated that white adipose tissue, representing the major portion of fat tissue and the major site for energy storage, contributes ~25 % of the circulating IL-6 in the absence of acute inflammation [159,165,166].

IL-6 plasma levels also correlate with the development of the metabolic syndrome [167] and predict future risk for type 2 diabetes [168,169]. It is important to note that these studies show an association with type 2 diabetes, not causality. It has been suggested that association between IL-6 and progression to type 2 diabetes may reflect an attempt to counter-regulate the low-grade inflammation induced by other inflammatory mediators such as TNFα [170]. An increase in adipose tissue mass is associated with insulin resistance, hyperglycemia, dyslipidemia, and hypertension – all components of the metabolic syndrome. IL-6 gene expression is related to adipose cell size [171], and IL-6 plasma concentration increases postprandially [172]. The role of IL-6 in insulin resistance remains controversial [170,173]. Nevertheless, human and experimental animal studies do suggest that IL-6 is involved in the development of insulin resistance in adipose tissue, skeletal muscle, and particularly in hepatocytes [160,170,174]. While the mechanism remains unclear, cytokines such as IL-6 and TNFα are able to decrease insulin action [175-177].

Perivascular adipose tissue increasingly is recognized as an important source of adipokines and proinflammatory cytokines, including IL-6 [178-180], but its role in cardiovascular disease remains unclear [181]. Perivascular adipose tissue plays a role in the regulation of arterial tone since it has been reported that adventitial adipose tissue attenuates responsiveness of rat aortic rings to phenylephrine and norepinephrine [182]. The identity of this perivascular fat-derived, vascular relaxing factor remains unknown, although leptin and/or adiponectin have been proposed. Nevertheless, these observations are intriguing and suggest that perivascular fat may have beneficial, protective effects under normal physiological conditions, but “perivascular adipose tissue dysfunction” may be deleterious under disease conditions such as obesity and diabetes [181].

## SUMMARY

In summary, IL-6 is a major indicator of significant cardiovascular disease of diverse etiologies and has emerged as a multi-faceted regulator of vascular tone and cellular inflammation. In this review, we have illustrated its complex mechanisms of signaling, mediated by classic membrane receptor or trans-signaling modalities, which act in concert to promote the targets and spectrum of IL-6 effects. Recent advances in understanding the molecular signaling pathway initiated by IL-6 through the STAT3 transcription factor has led to the discovery of novel coactivators required for STAT3 genomic effects that may be targets for vascular therapies. From this work it is clear that IL-6 has diverse actions including modulating endothelial-dependent vasorelaxation, monocyte differentiation, platelet function, procoagulant state, myocardial hypertrophy, and effects on obesity and intermediary metabolism. These studies underscore the central relevance of the IL-6-gp130 signaling pathway in vascular pathologies.
